# 1537. Influenza-Associated Hospitalization Rates and Proportion of Hospitalizations with Influenza and SARS-CoV-2 Coinfection, FluSurv-NET, October 1, 2021–April 23, 2022

**DOI:** 10.1093/ofid/ofac492.092

**Published:** 2022-12-15

**Authors:** Shikha Garg, Charisse N Cummings, Dawud Ujamaa, Pam Daily Kirley, Nisha B Alden, Maria Correa, Evan J Anderson, Andy Weigel, Maya Monroe, Val Tellez Nunez, Melissa McMahon, Susan L Ropp, Nancy L Spina, Maria Gaitan, Eli Shiltz, Melissa Sutton, Keipp Talbot, Melanie Crossland, Carrie Reed

**Affiliations:** Centers for Disease Control and Prevention, Atlanta, Georgia; Centers for Disease Control and Prevention, Atlanta, Georgia; Centers for Disease Control and Prevention, Atlanta, Georgia; California Emerging Infections Program, Oakland, California; Colorado Department of Public Health and Environment, Denver, Colorado; Connecticut Emerging Infections Program, Yale School of Public Health, New Haven, Connecticut; Emory University School of Medicine, Atlanta, Georgia; Iowa Department of Public Health, Des Moines, Iowa; Maryland Department of Health, Baltimore, Maryland; Michigan Department of Health and Human Services, Lansing, Michigan; Minnesota Department of Health, St. Paul, Minnesota; New Mexico Department of Health, Santa Fe, New Mexico; New York State Department of Health, Albany, New York; Rochester Emerging Infections Program, University of Rochester Medical Center, Rochester, New York; Ohio Department of Health, Columbus, Ohio; Oregon Health Authority, Portland, Oregon; Vanderbilt University Medical Center, Nashville, Tennessee; Salt Lake County Health Department, Salt Lake City, Utah; Centers for Disease Control and Prevention, Atlanta, Georgia

## Abstract

**Background:**

Influenza-associated hospitalization rates were low during the 2020–21 season. We describe influenza-associated hospitalization rates and prevalence of influenza and SARS-CoV-2 coinfection among patients hospitalized with influenza during 2021–22.

**Methods:**

We used data from the Influenza Hospitalization Surveillance Network (FluSurv-NET), a population-based surveillance system for laboratory-confirmed influenza-associated hospitalizations active from October—April of each year. We calculated cumulative and weekly hospitalization rates per 100,000 population and compared preliminary rates during 2021–22 with prior season rates (2010–11 through 2020–21). We determined the proportion of influenza-associated hospitalizations with SARS-CoV-2 coinfection during 2021–22.

**Results:**

During October 1, 2021—April 23, 2022, 3,262 influenza-associated hospitalizations were reported to FluSurv-NET; the cumulative hospitalization rate of 11.1 was higher than 2011–12 and 2020–21 season rates, but lower than rates observed during all other seasons since 2010–11 (Figure 1A). After peaking in the week ending January 1, 2022 (MMWR week 52), weekly hospitalization rates declined until the week ending February 19, 2022 (MMWR week 7) when they began to rise modestly, similar to patterns observed during several prior seasons (Figure 1B). Among the 3,262 hospitalizations, 87 (2.7%) had SARS-CoV-2 coinfection; the prevalence by age group was as follows: 0–17 years 3.4%, 18–49 years 2.8%, 50–64 years 3.5%, 65–74 years 2.5%, ≥ 75 years 1.6%. Among the 3,262 influenza-associated hospitalizations, the prevalence of SARS-CoV-2 coinfection by month (October 2021–April 2022), respectively, was 11.4%, 2.5%, 2.6%, 8.9%, 3.4%, 0.8%, and 0.5%.

**Conclusion:**

SARS-CoV-2 coinfection was uncommon among patients hospitalized with influenza during 2021–22. Likely due to ongoing COVID-19 mitigation measures, the influenza-associated hospitalization rate during 2021–22 was lower than rates observed in most seasons in the decade preceding the COVID-19 pandemic. A late rise in weekly influenza hospitalization rates in 2021–22 might have been a result of relaxation of COVID-19 mitigation measures and/or a late season peak in influenza activity.

**Disclosures:**

**Evan J. Anderson, MD**, GSK: Advisor/Consultant|GSK: Grant/Research Support|Janssen: Advisor/Consultant|Janssen: Grant/Research Support|Kentucky Bioprocessing, Inc: Data Safety Monitoring Board|MedImmune: Grant/Research Support|Medscape: Advisor/Consultant|Merck: Grant/Research Support|Micron: Grant/Research Support|NIH: Funding from NIH to conduct clinical trials of Moderna and Janssen COVID-19 vaccines|PaxVax: Grant/Research Support|Pfizer: Advisor/Consultant|Pfizer: Grant/Research Support|Regeneron: Grant/Research Support|Sanofi Pasteur: Advisor/Consultant|Sanofi Pasteur: Grant/Research Support|Sanofi Pasteur: Data Adjudication and Data Safety Monitoring Boards|WCG and ACI Clinical: Data Adjudication Board **Maya Monroe, MPH**, CDC -Emerging Infections Program: Grant/Research Support.

